# Effects of reduced salinity on the photosynthetic characteristics and intracellular DMSP concentrations of the red coralline alga, *Lithothamnion glaciale*

**DOI:** 10.1007/s00227-015-2650-8

**Published:** 2015-03-22

**Authors:** Heidi L. Burdett, Angela D. Hatton, Nicholas A. Kamenos

**Affiliations:** 1 Scottish Oceans Institute, University of St Andrews, St Andrews, UK; 2 Department of Earth and Environmental Sciences, University of St Andrews, St Andrews, UK; 3 School of Geographical and Earth Sciences, University of Glasgow, Glasgow, UK; 4 Scottish Association for Marine Science, Oban, UK

## Abstract

Mid- to high-latitude fjordic coastal environments experience naturally variable salinity regimes. Climate projections suggest that freshwater input into the coastal ocean will increase in the future, exposing coastal organisms to further periods of reduced salinity. This study investigated the effect of low salinity on *Lithothamnion glaciale*, a red coralline alga found in mid- to high-latitude fjordic regions, during a 21-day experiment. Specific measurements included: the intracellular concentration of dimethylsulphoniopropionate (DMSP, an algal secondary metabolite and major precursor to the climatically active gas dimethylsulphide), pigment composition and photosynthetic characteristics. No significant difference in intracellular DMSP concentrations was observed between treatments, suggesting that the primary function for DMSP in *L. glaciale* is not as a compatible solute, perhaps favouring an antioxidant role . Photosynthetic parameters (including pigment composition) exhibited a mixed response, suggesting some degree of photosynthetic resilience to reduced salinity. This study provides evidence of intracellular mechanisms adopted by *L. glaciale* in response to reduced salinity. This has significant implications for the survival of *L. glaciale* under a projected freshening scenario and provides organism-level detail to ecosystem-level projected changes should lower-salinity conditions become more frequent and more intense in the future.

## Introduction

Atmospheric CO_2_ concentrations have increased from ~280 parts per million (ppm) before the Industrial Revolution (IPCC 2013) to current levels of >395 ppm (NOAA 2014). Arguably, the higher levels of CO_2_ have already led to measurable changes in atmospheric processes such as elevated temperature (IPCC 2013) and increased storm activity (Mann and Emanuel 2006). A 270 % net increase in precipitation-derived freshwater input into the North Atlantic was observed during the 1960–1990s (Josey and Marsh 2005; Bindoff et al. 2007), stimulating a debate into the future evolution of marine salinity given atmospheric CO_2_ projections. By the year 2100, atmospheric temperature is projected to rise by up to 6 °C (IPCC 2013), increasing the ‘moisture-holding capacity’ of the atmosphere (Trenberth et al. 2007), and thus the potential for precipitation events, enhancing freshwater run-off into the coastal zone (Gillibrand et al. 2005), particularly in the mid/high latitudes (IPCC 2013). A rise in atmospheric temperatures may also lead to more pronounced seasonal ice melt (Hanna et al. 2008); run-off from the Greenland Ice Sheet into the Kangerlussaq drainage basin has increased by 113 km^3^ over the last 50 years (Hanna et al. 2008). Coralline algal climate proxies from Søndre Strømfjord, western Greenland, have shown that this has resulted in a reduction in average coastal salinity by ~5 units (Kamenos et al. 2012). Fjordic landscapes are also typically characterised by a variable salinity regime: Søndre Strømfjord has a freshwater-influenced surface layer in the upper 50–75 m of the water column; the salinity of the upper 20 m is <10 in late summer, because of ice melt (Nielsen et al. 2010). Similarly, in Loch Linnhe (western Scotland, 56.5°N), a low-salinity (~11) layer penetrates to ~10 m depth during the winter, increasing in depth through the spring as freshwater riverine discharge increases (Allen and Simpson 1998).

Non-geniculate red coralline algae (Rhodophyta: Corallinales) are widespread throughout the world’s coastal oceans, from the intertidal zone to >250 m depth (Foster 2001). These algae can grow as individual, unattached thalli known as maerl or rhodoliths, or may form an encrusting layer on bed rock (Foster 2001). Red coralline algae are ecologically (Kamenos et al. 2004a, b, c) and structurally (Tierney and Johnson 2012) important components of many coastal habitats and are often used as high-resolution palaeoclimatic proxies (Kamenos et al. 2008a; Kamenos 2010; Burdett et al. 2011). *Lithothamnion glaciale*, a key ecosystem engineer for these habitats, is found in mid- to high-latitude fjordic shallow-water environments (from 55 to 80°N, <20 m depth) (Foster 2001; Teichert et al. 2012) and thus is likely to have developed intracellular mechanisms to cope with changing osmotic conditions.

Thus, the survival of coralline algal habitats in variable coastal environments depends, in part, on an organisms’ response to periodic exposure to reduced salinity conditions. One possible mechanism may be through the production and regulation of compounds which can act as osmoprotectants (compatible solutes), such as dimethylsulphoniopropionate (DMSP) (Kirst 1996). DMSP can be produced by a range of marine algae and has been proposed to serve numerous cellular functions within algal cells, such as a compatible solute (Kirst 1996), an antioxidant (Sunda et al. 2002), a cryoprotectant (Karsten et al. 1996) and a grazing deterrent (Van Alstyne et al. 2001). The majority of studies investigating DMSP’s proposed compatible solute function have focussed on hyper-salinity (resulting in increased intracellular DMSP concentrations, e.g. Karsten et al. 1992). However, in mid- to high-latitude coastal systems, a hypo-salinity scenario is more ecologically relevant because of ice melt and land run-off. In general, it is thought that DMSP is regulated in response to longer-term, chronic changes in salinity (Edwards et al. 1988; Kirst 1989), rather than as a short-term ‘stress’ response, perhaps due to the energy outlay required to instigate changes in intracellular DMSP concentrations (Yoch 2002). In support of this, short-term hypo-salinity conditions do not appear to result in significant declines in intracellular DMSP in the macroalga *Ulva lactuca* (Van Alstyne et al. 2003; Ross and Alstyne 2007). Red coralline algae typically maintain high intracellular DMSP concentrations (Kamenos et al. 2008b). The effect of reduced salinity on intracellular DMSP concentrations in these organisms has not yet been investigated, but previous studies have shown a response to periodic changes in other environmental variables such as light (Rix et al. 2012; Burdett et al. 2014) or *p*CO2 (Burdett et al. 2012a, 2013). Regulation of intracellular DMSP concentrations may also depend on photosynthetic activity, as the precursor to DMSP, methionine, is an indirect product of photosynthesis (Wirtz and Droux 2005) and is an essential component of photosynthetic proteins such as RuBisCO and the D1-protein (Wirtz and Droux 2005).

Reductions in salinity can also have major implications for photosynthesis, particularly in the red algae (Larsen and Sand-Jensen 2006). The photosynthetic efficiency (*F*_v_/*F*_m_) of *L. glaciale* was reduced after 5-week exposure to a salinity of 3 (Wilson et al. 2004); however, prolonged periods at such low salinities are unlikely to regularly occur in situ. In brown algae, a reduction in photosynthetic efficiency has been observed in the microscopic life stages (zoospores) of the Arctic kelp *Alaria esculenta*, but not during adult life stages when exposed to a salinity of 20 (Fredersdorf et al. 2009). However, following prolonged exposure to low-salinity conditions, higher tolerance thresholds may develop. Optimal salinities for maximum electron transport rate and relative growth rate were higher in *Fucus vesiculosus* from Ireland (ambient salinity of 35, optimal salinity = 20–35), when compared to the same species in the Baltic Sea (ambient salinity of 5, optimal salinity = 10–20) (Nygård and Dring 2008). The physiology of the red alga *Gelidium coulteri* appeared to at least partially recover after a 5-week exposure to a low-salinity environment, despite initial decreases in photosynthetic parameters and initial increases in respiration (Macler 1988).

This study investigated the impact of reduced salinity on the intracellular DMSP concentration, pigment composition and photosynthetic characteristics of *L. glaciale*. It was hypothesised that, following prolonged exposure to reduced salinity conditions, DMSP concentrations would decrease (supporting the proposed compatible solute function) and photosynthetic characteristics would not ultimately be affected (indicating a degree of tolerance).

## Materials and methods

### Specimen collection

Free-living *Lithothamnion glaciale* thalli were collected from Loch Sween on the west coast of Scotland, UK (56°01.99′N, 05°36.13′W), in the summer of 2011 using SCUBA from a depth of 5 m. The west coast of Scotland is characterised by a typical post-glacial landscape, with steep valleys, thin soils and narrow fjords. Thalli were transported to the University of Glasgow in seawater at ambient temperature (12 °C), salinity (32) and light (40 µmol photons m^−2^ s^−1^). Thalli were transferred to 120-litre (0.80 × 0.35 × 0.40 m) re-circulating seawater tanks also maintained at ambient conditions of temperature (12 °C), light (40 µmol photons m^−2^ s^−1^, 16:8 h light: dark cycle) and salinity (32). Thalli were acclimated to laboratory conditions for 10 days before the experiment began. Light and water temperature followed natural field conditions as they would otherwise be additional confounding factors within the experiment.

### Experimental set-up

Three salinity treatments were used to assess the effect of chronic reductions in salinity: control (salinity = 32.1 ± 1.1, mean ± SD), low (21.5 ± 0.6, representative of precipitation run-off into a fjord; Allen and Simpson 1998) and very low (11.7 ± 0.9, representative of late summer ice melt into a fjord; Nielsen et al. 2010). A nested experimental design was adopted: three tanks (120 l volume) were used per treatment, each containing 50 thalli. Individuals were sampled at only one timepoint; thus, each timepoint is composed of samples independent from other timepoints. As with the acclimation, light (40 µmol photons m^−2^ s^−1^, 16:8 h light: dark cycle) and water temperature (12 °C) followed natural field conditions.

Water changes (25 %) were performed every 2 days throughout the experimental period to maintain water quality, and a constant water flow was maintained (circulation rate: 450 l h^−1^). Seawater was made from artificial sea salt (TropicMarin Pro Reef sea salt) according to manufacturer’s instructions. In the low-salinity treatments, the amount of salt in the water stock for water changes was reduced accordingly. Salinity in the treatment groups was gradually reduced during the first 7 days and maintained at the treatment level for another 14 days (total experimental period: 21 days). The salinity of the treatment tanks and water stock for water changes was monitored using a YSI Pro2030 conductivity probe (temperature compensated). This method of salinity reduction may also have reduced the level of nutrients in the treatment aquaria.

### Intracellular DMSP

Algal branches were sampled for intracellular DMSP at 0, 3, 7, 14 and 21 days. Ten branches from 10 individuals were sampled from each treatment tank at each timepoint, providing 30 branches per treatment, per timepoint. Each branch was gently cleaned with a soft brush before storage in 2 ml of 10 M sodium hydroxide in 14-ml glass vials. Vials were immediately crimped shut with gas-tight Pharma-Fix septa (Grace Alltech) to hydrolyse cellular DMSP to the gas dimethylsulphide. This method may yield DMS from other tertiary sulphonium compounds (e.g. Gage et al. 1997), but it is widely assumed that DMSP is the primary source of DMS (Van Alstyne and Puglisi 2007). Samples were incubated in the dark for 48 h before analysis. Intracellular DMSP (as dimethylsulphide) was quantified using gas chromatography (Shimadzu GC-2014 gas chromatograph) equipped with a flame photometric detector (200 °C, hydrogen gas pressure: 5.1 psi, air gas pressure 15.2 psi) and capillary column (5 % diphenyl–95 % dimethyl polysiloxane; length 25 m; inner diameter 0.25 mm; film thickness 0.25 µm, 45 °C). Samples were analysed by direct injection of the vial headspace (100 µl) into the GC (injector temperature: 45 °C; nitrogen carrier gas; total flow: 38.6 ml min^−1^). Concentrations were calibrated against DMSP standards (DMSP obtained from Research Plus Inc., Barnegat, USA). The standard and sample detection limit was 30 nmol of sulphur per injection; sample and standard precision was within 1 %. Results are presented as µM DMSP g^−1^ biomass to aid in comparison with fleshy macroalgae; the biomass of *L. glaciale* is ~3.50 % of the total fresh mass of the thallus (Burdett et al. 2012a).

### Pigment composition

The reflectance spectra of *L. glaciale* branches (*n* = 10 per treatment, randomly chosen from each of the three replicate treatment tanks) were measured at the beginning (day 0) and again end of the experiment (day 21, same individuals, but different branches to minimise repeat sampling errors) using a USB 2000+ Ocean Optics spectrometer following the protocol outlined in Burdett et al. (2014). Light (Arcadia T5 Marine White, 24 W) was directed at the algal branch via a 5-mm fibre optic probe (Walz GmbH, Effeltrich, Germany). Reflected light was transmitted to the spectrometer via a 400-µm single-fibre optic probe (Ocean Optics). Due to the small diameter and nonlinearity of *L. glaciale* branches, it was logistically difficult to maintain a fixed angle between the two fibre optic probes. Thus, for each sample, the probes were positioned to achieve maximum reflectance output based on the real-time spectrometer trace (Burdett et al. 2014). Percentage reflectance was calculated by comparison with a white standard (0 % absorbance across the whole spectra). The wavelengths of pigment absorbance were obtained from Hedley and Mumby (2002).

### Photosynthetic characteristics

Chlorophyll-*a* fluorescence measurements were conducted using a Diving-PAM fluorometer (Walz GmbH) and used to calculate photosynthetic characteristics of the algal thalli. Measurements were taken following the methodology outlined, and notation described, in Burdett et al. (2012b). A 5-mm-diameter fibre optic probe was used for all measurements, positioned 10 mm from branch tips. This approach maximises the signal-to-noise ratio and provides fluorescence data integrated over the whole branch length; along-branch heterogeneity has previously been observed in *L. glaciale* (Burdett et al. 2012b).

### Rapid light curves (RLCs)

Rapid light curves (RLCs), where organisms are exposed to pulses of saturating actinic light interspersed with 10–20 s of increasing levels of irradiance, have become well established within PAM fluorometry (Ralph and Gademann 2005). RLCs provide information on energy dissipation from light-limiting through to light-saturating conditions. However, due to the short exposure time at each irradiance step, steady-state conditions are not achieved during RLCs (Ralph and Gademann 2005). Thus, in contrast to traditional light curves, results from RLCs reflect actual, rather than optimal, photosynthetic state (Ralph and Gademann 2005).

RLCs (*n* = 15 per timepoint, per treatment) were conducted at 0, 3, 7, 14 and 21 days using eight irradiance steps ranging from 2 to 997 µmol photons m^−2^ s^−1^. Thalli were dark acclimated for 5 min in the experimental tanks prior to running the RLCs, which is sufficient time to induce full dark acclimation in *L. glaciale* (Burdett et al. 2012b). Each RLC produced a series of effective quantum efficiency measurements $\left( {F_{q}^{\prime } /F_{m}^{\prime } } \right)$ that were fitted to a nonlinear least squares regression model to describe the light response of quantum efficiency (Hennige et al. 2008; Burdett et al. 2012b):

1 $$F_{q}^{\prime } /F_{m}^{\prime } \, = \, \left[ {\left( {F_{q}^{\prime } /F_{m\;\;\hbox{max} }^{\prime } \times E_{k} } \right)\left( {1 \, {-} \, \exp \, \left( {{-}E/E_{k} } \right)} \right)} \right] \, / \, E$$

where *E*_*k*_ is the minimum saturation intensity (µmol photons m^−2^ s^−1^)^59^—the irradiance level where light shifts from being photosynthetically limiting to photosynthetically saturating. *E* is equivalent to photosynthetically active radiation (PAR, µmol photons m^−2^ s^−1^). For the first step of the RLC (where the algae were dark acclimated), *F*_v_/*F*_m_ was used instead of $F_{q}^{\prime } /F_{m}^{\prime }$. Equation 1 was also used to calculate the theoretical maximum effective quantum efficiency, $F_{q}^{\prime } /F_{m\;\;\hbox{max} }^{\prime }$. As $F_{q}^{\prime } /F_{m\;\;\hbox{max} }^{\prime }$ was derived from the RLC illumination, differences observed represent differences in light acclimation rather than environmental light availability (Suggett et al. 2007).

Electron transport rate (ETR, µmol electrons m^−2^ s^−1^) was also calculated from $F_{q}^{\prime } /F_{m}^{\prime }$ measurements at each actinic light intensity (*E*) of the RLC:

2 $${\text{ETR }} = F_{q}^{\prime } /F_{m}^{\prime } \, \times {\text{PAR}} \times 0.15 \times A$$

where PAR is the RLC irradiance (µmol photons m^−2^ s^−1^), 0.15 is a multiplication factor to take into account that 15 % of chlorophyll-*a* in red algae is associated with PSII (Goldstein et al. 1992; Figueroa et al. 2003; Burdett et al. 2012b) and *A* is the corrected total algal absorbance. For the first step of the RLC (where the algae were dark acclimated), *F*_v_/*F*_m_ was used instead of $F_{q}^{\prime } /F_{m}^{\prime }$. The maximum obtained ETR (ETR_MO_) values from the RLCs are presented.

Absorbance values (*A*) were calculated from the average absorbance of thalli branches between 400 and 700 nm (= range of PAR), determined from the spectra obtained from the pigment composition analysis. Average absorbance was corrected for non-pigment absorption by subtracting the average absorbance between 725 and 750 nm. The fraction of light absorbed by photosynthetic pigments (*A*) was calculated following Schubert et al. (2011):

3 $$A = \, 1 \, {-} \, 10^{ - D}$$

where *D* is the corrected absorbance between 400 and 700 nm.

### Statistics

Due to the nested experimental design, the mean of each response metric from each tank on day 21 was calculated for statistical comparisons (achieving *n* = 3 per treatment level). Neither DMSP nor photosynthetic parameter data could be transformed to meet parametric test assumptions; thus, a Kruskal–Wallis nonparametric test was used to investigate differences between treatments at the end of the experiment. Paired t-tests were used to compare reflectance spectra from each treatment at the start and end of the experiment (test assumptions met). All statistical analyses were conducted in Minitab version 15.

## Results

### Intracellular DMSP

**Fig. 1:**
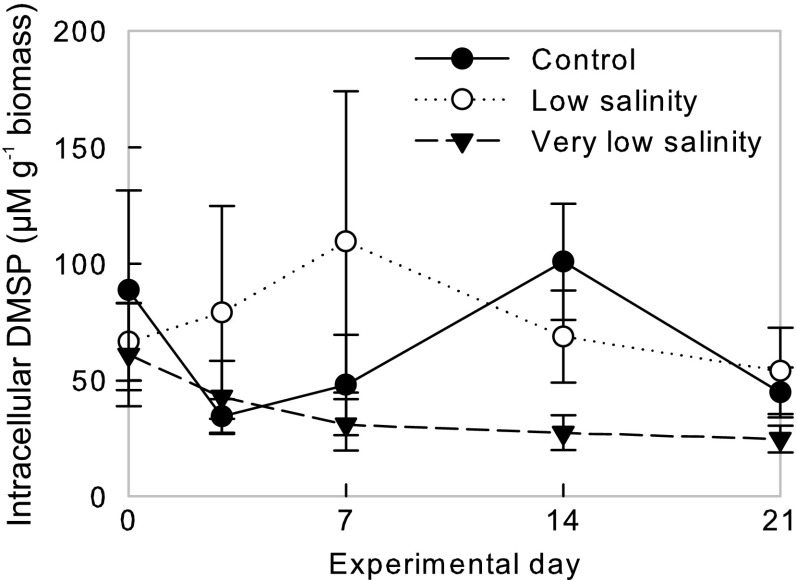
Intracellular DMSP concentrations (µmol g^−1^) of *Lithothamnion glaciale.* Thalli were maintained under control (*black circles*), low (*open circles*) and very low (*black triangles*) salinities over a 21-day experimental period. Data presented as mean ± SE

The control (final salinity = 32) and low (final salinity = 22)-salinity treatments were characterised by a greater variability in intracellular DMSP concentrations than the very low-salinity treatment (final salinity = 12), particularly in the initial stages of the experiment (Fig. 1). Intracellular DMSP in the very low-salinity treatment was generally the lowest of the three treatments (~25–30 µM g^−1^ biomass from day 7, compared to >45 µM g^−1^ biomass for the low-salinity and control treatments), but was not significantly different to the control group by the end of the experiment (*H*_2_ = 0.62, *p* = 0.73, Fig. 1).

### Pigment composition

**Fig. 2:**
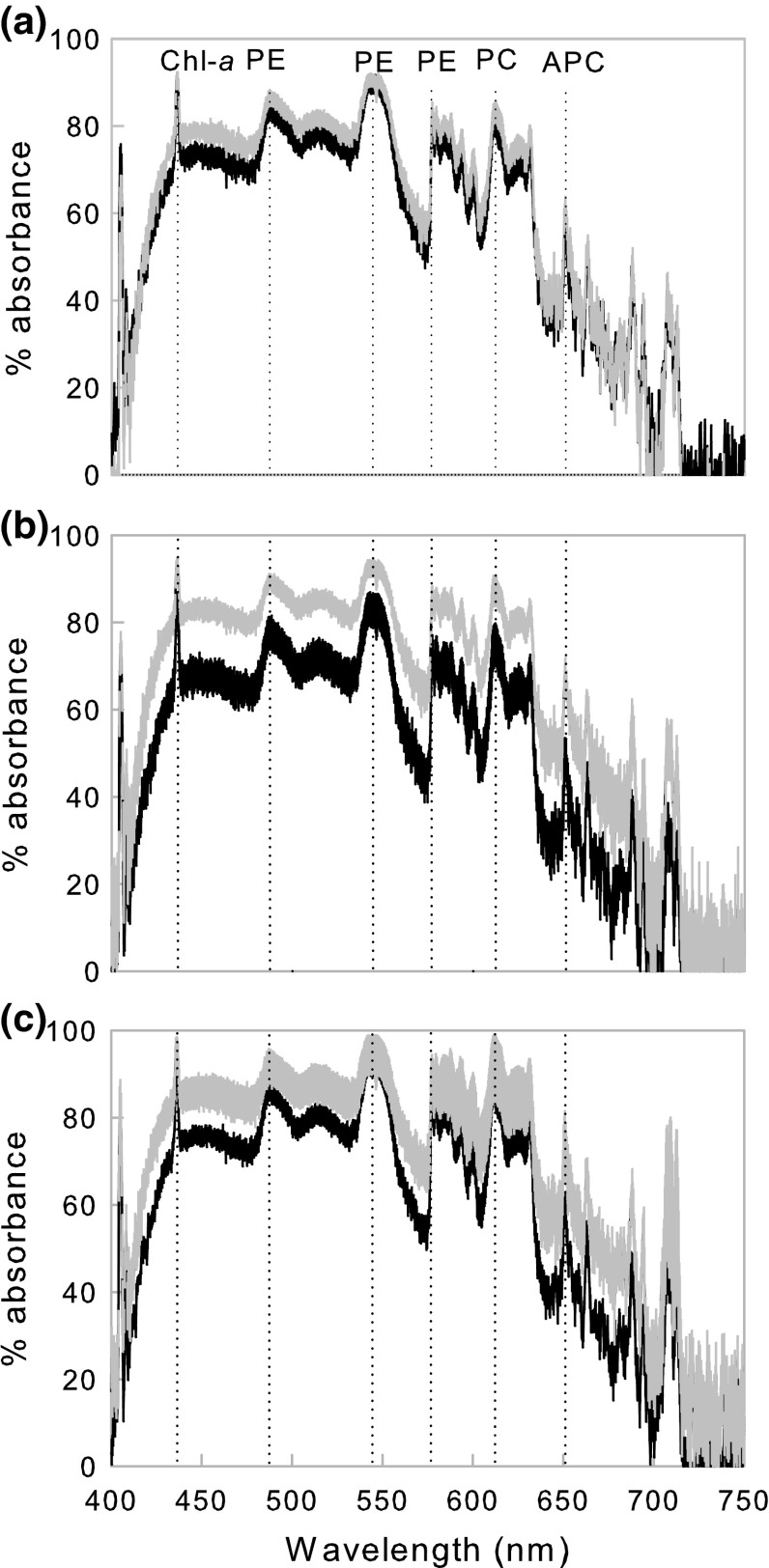
Absorbance spectra of *Lithothamnion glaciale*. Measurements taken at 0 (*black line*) and 21 days (*grey line*) from samples at **a** control, **b** low and **c** very low salinities. *Width of the line* represents mean ± SD. *Vertical dotted lines* indicate the absorption wavelength of Rhodophyta pigments: Chlorophyll-a (Chl-a), phycoerythrin (PE), phycocyanin (PC) and allophycocyanin (APC).  % absorbance is relative to a white standard, which yields 0 % absorbance across the whole spectra

The absorbance spectra from all treatments at 0 and 21 days were similar in peak and trough presence/absence, although the absolute % absorbance varied (Fig. 2). Peaks in absorbance were observed at wavelengths expected for known Rhodophyta pigments: Chlorophyll-a (435 nm), phycoerythrin (488, 546, 576 nm), phycocyanin (613 nm) and allophycocyanin (652 nm). No change in the absorbance spectra was observed between the beginning and end of the experiment in the control treatment (*T* = 1.54, *p* = 0.12; Fig. 2a). In contrast, a significant increase in % absorbance across the whole spectra (PAR range: 400–700 nm) was observed in the low (final salinity = 22, *T* = 95.64, *p* < 0.001)- and very low (final salinity = 12, *T* = 68.49, *p* < 0.001)-salinity treatments by day 21 (Fig. 2b,c); this was accompanied by a modest increase in variability between thalli (Fig. 2b,c) and fouling of the epithelial surface.

### Photosynthetic characteristics

**Fig. 3:**
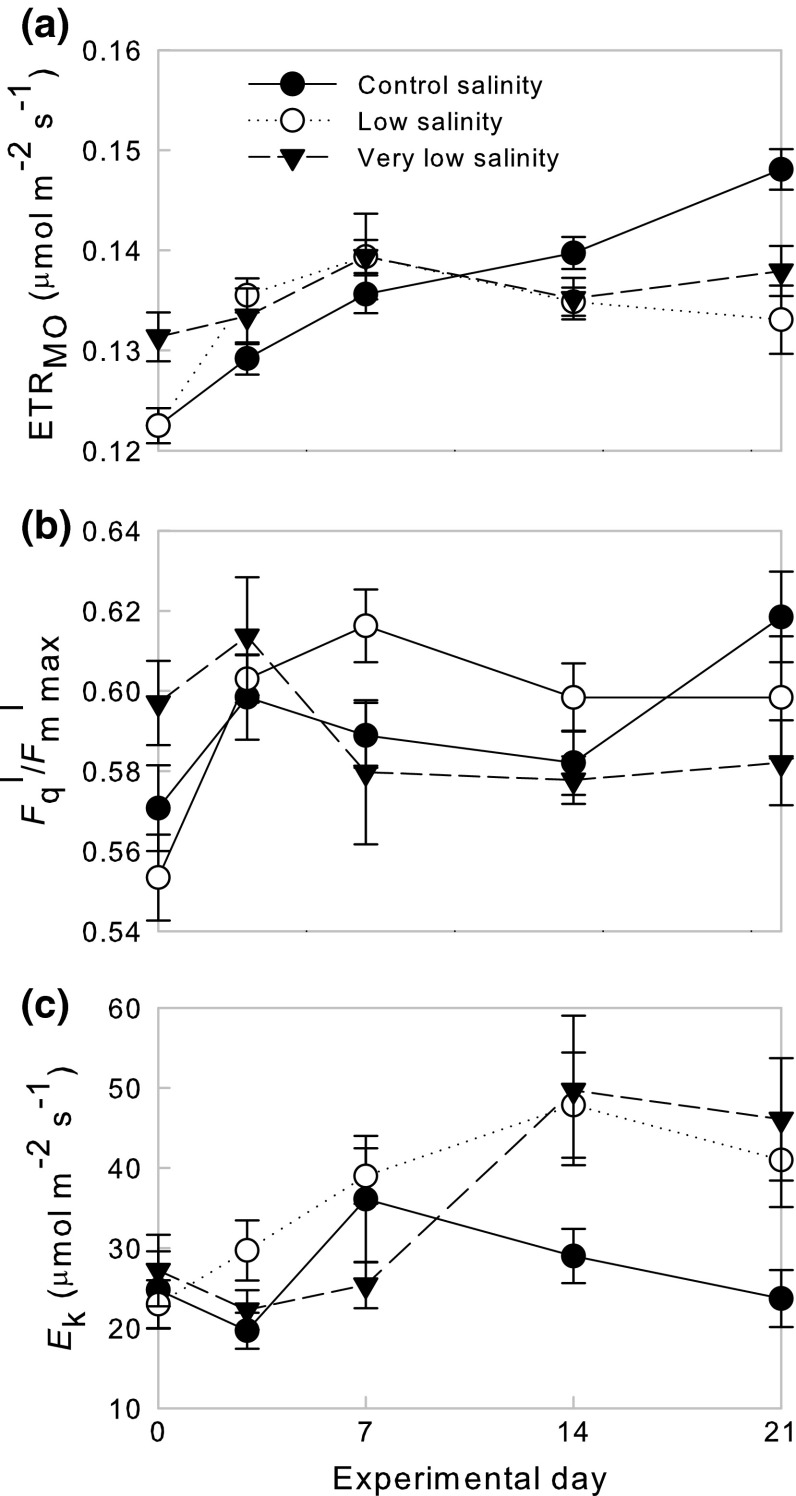
Photosynthetic characteristics of *Lithothamnion glaciale*. **a** Maximum obtained ETR (µmol electrons m^−2^ s^−1^), **b** $F_{q}^{\prime} /F_{m\,{\max} }^{\prime }$ and **c** *E*_*k*_ (µmol photons m^−2^ s^−1^) under control (*black circles*), low (*open circles*)- and very low (*black triangles*)-salinity conditions over a 21-day experimental period. Data presented as mean ± SE

ETR_MO_ in the control treatment (final salinity = 32) was significantly higher (0.15 ± 0.002 µmol electrons m^−2^ s^−1^, mean ± SE) than the low (final salinity = 22)- and very low (final salinity = 12)-salinity treatments (0.13 ± 0.003 and 0.14 ± 0.02 µmol electrons m^−2^ s^−1^, respectively, mean ± SE) by the end of the experiment (*H*_2_ = 12.62, *p* = 0.002, Fig. 3a), although the control group was characterised by a general increase in ETR_MO_ over the course of the experiment (day 0: 0.12 ± 0.002, day 21: 0.15 ± 0.002 µmol electrons m^−2^ s^−1^, mean ± SE, Fig. 3a). In contrast, no significant difference in $F_{q}^{\prime } /F_{m\;\;\hbox{max} }^{\prime }$ was observed between treatments at the end of the experiment, which remained between 0.55 and 0.62 throughout the experimental period (*H*_2_ = 5.48, *p* = 0.064, Fig. 3b). A significant difference in *E*_*k*_ was also observed between treatments at the end of the experiment (*H*_2_ = 7.81, *p* = 0.020, Fig. 3c): in the control and low-salinity treatments, *E*_*k*_ rose to a maximum of ~45 µmol photons m^−2^ s^−1^ on day 14, whilst *E*_*k*_ in the very low-salinity treatment (final salinity = 12) remained relatively constant (~25 photons µmol m^−2^ s^−1^) throughout the experiment (Fig. 3c).

## Discussion

Climate projections suggest that ice melt and high-intensity storm activity will increase in the future (Hanna et al. 2008; Knutson et al. 2010), increasing the input of freshwater into the coastal zone and exposing coastal organisms to prolonged, more frequent, periods of reduced salinity. This study has shown that the bed-forming red coralline alga *Lithothamnion glaciale*, an integral species in many temperate and polar coastal habitats, harbours intracellular mechanisms that enable it to tolerate a periodic reduction in salinity.

Intracellular DMSP concentrations of *L. glaciale* (normalised to biomass) were comparable to other high DMSP macroalgae such as the Ulvales (Van Alstyne and Puglisi 2007). DMSP is known to act as a compatible solute in marine algae, helping to protect against external changes in salinity. DMSP did not significantly decline under hypo-salinity conditions, supporting previous studies of the green macroalga *Ulva lactuca* (Van Alstyne et al. 2003; Ross and Alstyne 2007), and suggesting that other carbohydrate molecules are regulated in response to hypo-salinity (e.g. glycine betaine). These results may also have been confounded by the reduction in nutrients: DMSP can replace the role of N-containing osmolytes (e.g. proline) (Stefels 2000) and thus may increase during nutrient limitation (Stefels 2000). However, this has not been universally observed; some macroalgae exhibit no response to varying nitrogen conditions (Van Alstyne et al. 2007). This suggests that osmotic control may not be the priority function for DMSP in *L. glaciale*, even when exposed to a reduced salinity environment. However, a range of evidence is available which suggests the proposed antioxidant function for DMSP (Sunda et al. 2002) is active in red coralline algae—*L. glaciale* is often light-saturated even under ambient conditions (Burdett et al. 2012b), and intracellular DMSP concentrations are regulated in response to varying light levels (Rix et al. 2012; Burdett et al. 2014). The large variability in intracellular DMSP concentrations may also be influenced by the antioxidant function: ‘self-shading’ by outer branches results in photosynthetic heterogeneity (Burdett et al. 2012b), and likely heterogeneity in antioxidant requirements, which may be expressed as intra-thallus heterogeneity in intracellular DMSP concentrations.

Despite the observed epithelial fouling, no change in Rhodophyta-specific pigment composition (phycoerythrin, phycocyanin and allophycocyanin) was observed at the end of the 21-day experiment, providing confidence that epiphytic micro-organisms (e.g. cyanobacteria) did not confound the results. This is in contrast to other red macroalgae (e.g. *Gelidium coulteri*, Macler 1988) and suggests that *L. glaciale* pigmentation is more robust to reduced salinity than other red macroalgae. However, some evidence for dynamic photoinhibition in response to salinity reduction was observed, as an increase in the overall absorbance of *L. glaciale* thalli in the low- and very low-salinity treatments. This may have affected the alga’s light-harvesting capacity and supports the observed reduction in *E*_*k*_. Absorbance data may have also been affected by the epithelial fouling; branches were not cleaned of fouling material prior to conducting the spectral analysis. However, as Rhodophyta pigments were still easily detected, this was not considered a major artefact. Dynamic photoinhibition has also been observed in other temperate macroalgae (Edwards and Kim 2010), tropical red coralline algae (Burdett et al. 2014) and seagrass plants (Belshe et al. 2007); such mechanisms may be critical for survival in naturally variable coastal environments.

Photosynthetic characteristics of the control treatment thalli were within the range observed by other laboratory and field PAM studies on *L. glaciale* (Burdett et al. 2012b). PAM fluorometry does not provide direct measurements of photosynthetic output (e.g. oxygen production) so PAM-derived ETR may be more indicative of photosynthetic capacity rather than actual photosynthetic rate (Enríquez and Borowitzka 2010). However, ETR values derived from PAM techniques can correspond well to photosynthesis rate, particularly at low irradiances (Figueroa et al. 2003; Nielsen and Nielsen 2008). $F_{q}^{\prime } /F_{m\;\;\hbox{max} }^{\prime }$ in all treatments was comparable to that observed for *L. glaciale* thalli in the field (Burdett et al. 2012b). This indicates that photosynthetic mechanisms were not severely affected by reduced salinity, further highlighting the tolerance of *L. glaciale* to reduced salinity conditions compared with other red macroalgae.

The gradual, but modest, increase in ETR in the control treatment may have been caused by the static light regime of laboratory conditions and highlights the moderate impact of reduced salinity on the photosynthetic characteristics of *L. glaciale*. By day 14, the *E*_*k*_ of thalli in the control and low-salinity treatments was similar to the experimental conditions (40 µmol photons m^−2^ s^−1^) and to that observed for *L. glaciale* in the field (Burdett et al. 2012b), indicating that (1) full acclimation to the static laboratory environment took 3–4 weeks and should be taken into account in future red coralline algal studies and (2) moderate decreases in salinity did not affect the optimal irradiance required for photosynthesis in *L. glaciale*. Continued acclimation to the laboratory conditions during the beginning of the experiment is also indicated by the relatively large variation in intracellular DMSP concentrations for the first 7 days. Pre-experimentation acclimation periods are typically <10 days; this study shows that a prolonged acclimation period is a necessary consideration in red coralline algal studies. In the very low-salinity treatment, *E*_k_ remained lower than the experimental irradiance level of 40 µmol photons m^−2^ s^−1^. Thus, at this irradiance level, the algae will have been exposed to light-saturating conditions, increasing the likelihood of oxidant production and photodamage, reinforcing the proposed antioxidant function for DMSP in red coralline algae.

Despite low numbers of truly individual replicates (*n* = 3 experimental tanks with a nested design), at the organism level, this study suggests that *L. glaciale* may be able to survive periodic freshening events, although repeated exposure may compromise its survival by permitting excessive fouling on the alga’s surface and reducing its antioxidant capacity. During prolonged exposure to reduced salinity conditions, the photosynthetic apparatus remained operational, and it appeared that only marginal regulation of intracellular metabolites was required in response to the hypo-salinity regime. The results of this study also have broader, ecosystem-level, implications. Juvenile invertebrates such as the queen scallop *Aequipecten opercularis* (Kamenos et al. 2004b, c) preferentially settle on live *L. glaciale* beds and tropical red coralline algae provide important settlement cues for invertebrate larvae (Huggett et al. 2006; Steller and Cáceres-Martinez 2009). The exact cues for settlement are unknown, but may be affected by changes in intracellular DMSP (Steinberg and De Nys 2002; Kiehn and Morris 2010) or surface fouling, affecting adult invertebrate recruitment rates and the subsequent development of reefal ecosystems. Lastly, ecosystem grazing dynamics may be mediated by algal-derived DMSP (Van Alstyne and Houser 2003) and thus may also be affected when intracellular DMSP concentrations are regulated in response to environmental drivers such as salinity reduction.

## References

[CR1] Allen GL, Simpson JH (1998). Deep water inflows to upper loch linnhe. Estuar Coast Shelf Sci.

[CR2] Belshe EF, Durako MJ, Blum JE (2007). Photosynthetic rapid light curves (RLC) of *Thalassia testudinum* exhibit diurnal variation. J Exp Mar Biol Ecol.

[CR3] Bindoff NL, Willebrand J, Artale V, Cazenave A, Gregory J, Gulev S, Hanawa K, Quéré CL, Levitus S, Nojiri Y, Shum CK, Talley LD, Unnikrishnan A, Solomon S, Qin D, Manning M, Chen Z, Marquis M, Averyt KB, Tignor M, Miller HL (2007). Observations: oceanic climate change and sea level. Climate change 2007: the physical science basis contribution of working group I to the fourth assessment report of the intergovernmental panel on climate change.

[CR4] Burdett H, Kamenos NA, Law A (2011). Using coralline algae to understand historic marine cloud cover. Palaeogr Palaeoclim Palaeoecol.

[CR5] Burdett HL, Aloisio E, Calosi P, Findlay HS, Widdicombe S, Hatton AD, Kamenos NA (2012). The effect of chronic and acute low pH on the intracellular DMSP production and epithelial cell morphology of red coralline algae. Mar Biol Res.

[CR6] Burdett HL, Hennige SJ, Francis FT-Y, Kamenos NA (2012). The photosynthetic characteristics of red coralline algae, determined using pulse amplitude modulation (PAM) fluorometry. Bot Mar.

[CR7] Burdett HL, Donohue PJC, Hatton AD, Alwany MA, Kamenos NA (2013). Spatiotemporal variability of dimethylsulphoniopropionate on a fringing coral reef: the role of reefal carbonate chemistry and environmental variability. PLoS ONE.

[CR8] Burdett HL, Keddie V, MacArthur N, McDowall L, McLeish J, Spielvogel E, Hatton AD, Kamenos NA (2014). Dynamic photoinhibition exhibited by red coralline algae in the Red Sea. BMC Plant Biol.

[CR9] Edwards MS, Kim KY (2010). Diurnal variation in relative photosynthetic performance in giant kelp *Macrocystis pyrifera* (Phaeophyceae, Laminariales) at different depths as estimated using PAM fluorometry. Aquat Bot.

[CR10] Edwards DM, Reed RH, Stewart WDP (1988). Osmoacclimation in *Enteromorpha intestinalis* long-term effects of osmotic stress on organic solute accumulation. Mar Biol.

[CR11] Enríquez S, Borowitzka MA, Suggett DJ, Prášil O, Borowitzka MA (2010). The use of the fluorescence signal in studies of seagrasses and macroalgae. Chlorophyll a fluorescence in aquatic sciences: methods and applications.

[CR12] Figueroa F, Conde-Álvarez R, Gómez I (2003). Relations between electron transport rates determined by pulse amplitude modulated chlorophyll fluorescence and oxygen evolution in macroalgae under different light conditions. Photosynth Res.

[CR13] Foster MS (2001). Rhodoliths: between rocks and soft places. J Phycol.

[CR14] Fredersdorf J, Müller R, Becker S, Wiencke C, Bischof K (2009). Interactive effects of radiation, temperature and salinity on different life history stages of the Arctic kelp Alaria esculenta (Phaeophyceae). Oecologia.

[CR15] Gage DA, Rhodes D, Nolte KD, Hicks WA, Leustek T, Cooper AJL, Hanson AD (1997). A new route for synthesis of dimethylsulphoniopropionate in marine algae. Nature.

[CR16] Gillibrand PA, Cage AG, Austin WEN (2005). A preliminary investigation of basin water response to climate forcing in a Scottish fjord: evaluating the influence of the NAO. Cont Shelf Res.

[CR17] Goldstein JI, Romig AD, Newbury DE, Lyman CE, Echlin P, Fiori C, Joy DC, Lifshin E (1992). Scanning electron microscopy and x-ray microanalysis.

[CR18] Hanna E, Huybrechts P, Steffen K, Cappelen J, Huff R, Shuman C, Irvine-Fynn T, Wise S, Griffiths M (2008). Increased runoff from melt from the Greenland ice sheet: a response to global warming. J Clim.

[CR19] Hedley JD, Mumby PJ (2002). Biological and remote sensing perspectives of pigmentation in coral reef organisms. Adv Mar Biol.

[CR20] Hennige S, Smith D, Perkins R, Consalvey M, Paterson D, Suggett D (2008). Photoacclimation, growth and distribution of massive coral species in clear and turbid waters. Mar Ecol Prog Ser.

[CR21] Huggett M, Williamson J, de Nys R, Kjelleberg S, Steinberg P (2006). Larval settlement of the common Australian sea urchin *Heliocidaris erythrogramma* in response to bacteria from the surface of coralline algae. Oecologia.

[CR22] IPCC (2013) Summary for policymakers working group I contribution to the IPCC fifth assessment report climate change 2013: the physical science basis

[CR23] Josey SA, Marsh R (2005). Surface freshwater flux variability and recent freshening of the North Atlantic in the eastern subpolar gyre. J Geophys Res.

[CR24] Kamenos NA (2010). North Atlantic summers have warmed more than winters since 1353 and the response of marine zooplankton. Proc Natl Acad Sci.

[CR25] Kamenos NA, Moore PG, Hall-Spencer J (2004). The small-scale distribution of juvenile gadoids in shallow inshore waters; what role does maerl play?. ICES J Mar Sci.

[CR26] Kamenos NA, Moore PG, Hall-Spencer JM (2004). Attachment of the juvenile queen scallop (*Aequipecten opercularis* (L.)) to maerl in mesocosm conditions; juvenile habitat selection. J Exp Mar Biol Ecol.

[CR27] Kamenos NA, Moore PG, Hall-Spencer JM (2004). Nursery-area function of maerl grounds for juvenile queen scallops *Aequipecten opercularis* and other invertebrates. Mar Ecol Prog Ser.

[CR28] Kamenos NA, Cusack M, Moore PG (2008). Coralline algae are global palaeothermometers with bi-weekly resolution. Geochim Cosmochim Acta.

[CR29] Kamenos NA, Strong SC, Shenoy DM, Wilson ST, Hatton AD, Moore PG (2008). Red coralline algae as a source of marine biogenic dimethylsulphoniopropionate. Mar Ecol Prog Ser.

[CR30] Kamenos NA, Hoey TB, Nienow P, Fallick AE, Claverie T (2012). Reconstructing Greenland ice sheet runoff using coralline algae. Geology.

[CR31] Karsten U, Kirst G, Wiencke C (1992). Dimethylsulphoniopropionate (DMSP) accumulation in green macroalgae from polar to temperate regions: interactive effects of light versus salinity and light versus temperature. Polar Biol.

[CR32] Karsten U, Kuck K, Vogt C, Kirst GO, Kiene RP, Visscher PT, Keller MD, Kirst GO (1996). Dimethylsulfoniopropionate production in phototrophic organisms and its physiological function as a cryoprotectant. Biological chemistry of DMSP and related sulfonium compounds.

[CR33] Kiehn WM, Morris JT (2010). Variability in dimethylsulfoniopropionate (DMSP) concentrations in *Spartina alterniflora* and the effect on *Littoraria irrorata*. Mar Ecol Prog Ser.

[CR34] Kirst G (1989). Salinity tolerance of eukaryotic marine algae. Annu Rev Plant Physiol Plant Mol Biol.

[CR35] Kirst G, Kiene RP, Visscher PT, Maureen Keller D, Kirst G (1996). Osmotic adjustment in phytoplankton and macroalgae: the use of dimethylsulfoniopropionate (DMSP). Biological and environmental chemistry of DMSP and related sulfonium compounds.

[CR36] Knutson TR, McBride JL, Chan J, Emanuel K, Holland G, Landsea C, Held I, Kossin JP, Srivastava AK, Sugi M (2010). Tropical cyclones and climate change. Nature Geosci.

[CR37] Larsen A, Sand-Jensen K (2006). Salt tolerance and distribution of estuarine benthic macroalgae in the Kattegat-Baltic Sea area. Phycologia.

[CR38] Macler BA (1988). Salinity effects on photosynthesis, carbon allocation, and nitrogen assimilation in the red alga, gelidium coulteri. Plant Physiol.

[CR39] Mann ME, Emanuel KA (2006). Atlantic hurricane trends linked to climate change. EOS Trans AGU.

[CR40] Nielsen H, Nielsen S (2008). Evaluation of imaging and conventional PAM as a measure of photosynthesis in thin- and thick-leaved marine macroalgae. Aquat Biol.

[CR41] Nielsen MH, Erbs-Hansen DR, Knudsen KL (2010). Water masses in Kangerlussuaq, a large fjord in West Greenland: the processes of formation and the associated foraminiferal fauna. Polar Res.

[CR42] NOAA (2014) Trends in atmospheric carbon dioxide

[CR43] Nygård CA, Dring MJ (2008). Influence of salinity, temperature, dissolved inorganic carbon and nutrient concentration on the photosynthesis and growth of Fucus vesiculosus from the Baltic and Irish Seas. Eur J Phycol.

[CR44] Ralph PJ, Gademann R (2005). Rapid light curves: a powerful tool to assess photosynthetic activity. Aquat Bot.

[CR45] Rix LN, Burdett HL, Kamenos NA (2012). Irradiance-mediated dimethylsulphoniopropionate (DMSP) responses of red coralline algae. Estuar Coast Shelf Sci.

[CR46] Ross C, Alstyne KLV (2007). Intraspecific variation in stress-induced hydrogen peroxide scavenging by the ulvoid macroalga *Ulva Lactuca*. J Phycol.

[CR47] Schubert N, Garcia-Mendoza E, Enriquez S (2011). Is the photo-acclimatory response of Rhodophyta conditioned by the species carotenoid profile?. Limnol Oceanogr.

[CR48] Stefels J (2000). Physiological aspects of the production and conversion of DMSP in marine algae and higher plants. J Sea Res.

[CR49] Steinberg PD, De Nys R (2002). Chemical Mediation of Colonization of Seaweed Surfaces. J Phycol.

[CR50] Steller D, Cáceres-Martinez C (2009). Coralline algal rhodoliths enhance larval settlement and early growth of the Pacific calico scallop *Argopecten ventricosus*. Mar Ecol Prog Ser.

[CR51] Suggett DJ, Le Floc’H E, Harris GN, Leonardos N, Geider RJ (2007). Different strategies of photoacclimation by two strains of *Emiliania huxleyi* (Haptophyta). J Phycol.

[CR52] Sunda W, Kieber DJ, Kiene RP, Huntsman S (2002). An antioxidant function for DMSP and DMS in marine algae. Nature.

[CR53] Teichert S, Woelkerling W, Rüggeberg A, Wisshak M, Piepenburg D, Meyerhöfer M, Form A, Büdenbender J, Freiwald A (2012). Rhodolith beds (Corallinales, Rhodophyta) and their physical and biological environment at 80°31′N in Nordkappbukta (Nordaustlandet, Svalbard Archipelago, Norway). Phycologia.

[CR54] Tierney PW, Johnson ME (2012). Stabilization role of crustose coralline algae during late pleistocene reef development on Isla cerralvo, Baja california Sur (Mexico). J Coast Res.

[CR55] Trenberth KE, Jones PD, Ambenje P, Bojariu R, Easterling D, Tank AK, Parker D, Rahimzadeh F, Renwick JA, Rusticucci M, Soden B, Zhai P, Solomon S, Qin D, Manning M, Chen Z, Marquis M, Averyt KB, Tignor M, Miller HL (2007). Observations: surface and atmospheric climate change. *Climate Change 2007: the physical science basis* contribution of working group I to the fourth assessment report of the intergovernmental panel on climate change.

[CR56] Van Alstyne KL, Houser LT (2003). Dimethylsulphide release during macroinvertebrate grazing and its role as an activated chemical defense. Mar Ecol Prog Ser.

[CR57] Van Alstyne K, Puglisi M (2007). DMSP in marine macroalgae and macroinvertebrates: distribution, function, and ecological impacts. Aquat Sci.

[CR58] Van Alstyne KL, Wolfe GV, Freidenburg TL, Neill A, Hicken C (2001). Activated defense systems in marine macroalgae: evidence for an ecological role for DMSP cleavage. Mar Ecol Prog Ser.

[CR59] Van Alstyne KL, Pelletreau K, Rosario K (2003) The Effects of Salinity on Dimethylsulfoniopropionate Production in the Green Alga Ulva fenestrata Postels et Ruprecht (Chlorophyta). Bot Mar 150: 350–356

[CR60] Van Alstyne K, Koellermeier L, Nelson T (2007). Spatial variation in dimethylsulfoniopropionate (DMSP) production in *Ulva lactuca* (Chlorophyta) from the Northeast Pacific. Mar Biol.

[CR61] Wilson S, Blake C, Berges JA, Maggs CA (2004). Environmental tolerances of free-living coralline algae (maerl): implications for European marine conservation. Biol Conserv.

[CR62] Wirtz M, Droux M (2005). Synthesis of the sulfur amino acids: cysteine and methionine. Photosynth Res.

[CR63] Yoch DC (2002). Dimethylsulfoniopropionate: its sources, role in the marine food web, and biological degradation to dimethylsulfide. Appl Environ Microbiol.

